# Genetic Characterization of Carbapenem-Resistant *Enterobacteriaceae* and the Spread of Carbapenem-Resistant *Klebsiella pneumonia* ST340 at a University Hospital in Thailand

**DOI:** 10.1371/journal.pone.0139116

**Published:** 2015-09-25

**Authors:** Thidarat Netikul, Pattarachai Kiratisin

**Affiliations:** Department of Microbiology, Faculty of Medicine Siriraj Hospital, Mahidol University, Bangkok, Thailand; University of Pittsburgh, UNITED STATES

## Abstract

Carbapenem-resistant *Enterobacteriaceae* (CRE) has increasingly spread worldwide in the past decade. The prevalence and characteristics of CRE in Thailand are unknown. In this study, we conducted a 2-year surveillance of CRE among 12,741 clinical isolates of *Enterobacteriaceae* at the largest university hospital in Thailand with molecular characterization of beta-lactamase (*bla*) genes, including carbapenemase genes. The CRE prevalence was 1.4%. *bla*_KPC-13_ and *bla*_IMP-14a_ were the only carbapenemase genes detected among these CRE isolates. *bla*_KPC-13_ gene was found in a single isolate of *Escherichia coli*, *Enterobacter cloacae* and *Citrobacter freundii*, and *bla*_IMP-14a_ was found in four isolates of *Klebsiella pneumoniae*. Carbapenem-resistant *K*. *pneumoniae* (CRKP) isolates were resistant to multiple carbapenems at a higher ratio than other CRE species, and thus were further characterized for resistance phenotypes, *bla* genotypes and molecular epidemiology. Most CRKP isolates harboured multiple *bla* genes, especially those related to extended-spectrum beta-lactamases. Seven CRKP isolates were resistant to all tested carbapenems, and showed decreased *ompK35* and/or *ompK36* porin gene expression. Molecular typing of CRKP based on pulsed-field gel electrophoresis (PFGE) demonstrated several unrelated clones. Multilocus sequence typing (MLST) was partially concordant with PFGE results and revealed that ST340, a member of drug-resistant *K*. *pneumoniae* clonal complex 258, was the most predominant clone, followed by ST48, ST11 and ST273. The novel ST1645 was identified from this study. ST340 has neither been shown to be predominated among CRKP from other studies, nor been reported in Thailand. Therefore, it emphases a critical concern to monitor and control the spread of CRKP.

## Introduction

Carbapenems are broad-spectrum beta-lactam agents frequently used in treatment of infections caused by multidrug resistant gram-negative bacteria. Due to a rise of extended-spectrum beta-lactamase (ESBL)-producing *Enterobacteriaceae* in many countries, carbapenems have been increasingly used as a common drug of choice [1]. *Enterobacteriaceae* that are resistant to carbapenems, known as carbapenem-resistant *Enterobacteriaceae* (CRE), have gradually emerged and thus strictly limiting options for treatment [2]. This is considered a serious threat because CRE are significantly associated with higher mortality rates [3], [4]. Production of various carbapenemases is a key mechanism mediating the emergence of CRE. *Klebsiella pneumoniae* carbapenemase (KPC), encoded by *bla*_KPC_ gene, is a particularly important enzyme related to treatment failure in serious infections and is produced by various species of *Enterobacteriaceae* [5–9]. Resistance to carbapenems among *Enterobacteriaceae* could also be mediated by other families of carbapenemases including NDM, IMP, VIM and OXA-48, in which outbreaks of *K*. *pneumoniae* producing these enzymes have been described [8], [10–13]. Other non-enzymatic mechanisms were also related to carbapenem resistance. For example, a decrease in expression of outer membrane proteins (OMPs), especially OmpK35 and OmpK36 porins, has been shown to be associated with increased MICs of both cephalosporins and carbapenems in *K*. *pneumoniae*, including outbreak strains [14], [15]. However, it may not be the major component of carbapenem resistance, i.e. only carbapenemase production without defective porin expression could strongly elevate carbapenem MICs [16], [17].

*K*. *pneumoniae* is known to be the most common species among CRE, hereafter called carbapenem-resistant *K*. *pneumoniae* (CRKP). CRKP has been reported in many countries and their molecular epidemiology based on multilocus sequence typing (MLST) demonstrates that various sequence types (STs) are widespread [18]. For example, *K*. *pneumoniae* ST258 is most commonly associated with carbapenem resistance in the United States and Greece [10], [19]. The ST258 clone belongs to the multidrug-resistant clonal complex (CC) 258 of *K*. *pneumoniae* that is commonly associated with KPC-producing *K*. *pneumoniae* strains. Interestingly, it has been reported that other members of CC258, especially ST11, has become predominant clones in disseminating both *bla*_KPC_ and *bla*_NDM_, or in association with carbapenem resistance, in Brazil and many Asian countries [20–24]. A recent survey in Russia also reported that ST340, another member of CC258, was associated with NDM-1-producing *K*. *pneumoniae* isolates [25]. Therefore, a rising trend of *K*. *pneumoniae* CC258 in the spread of CRE is worthwhile for further investigation.

In Thailand, an overall prevalence of ESBL-producing *K*. *pneumoniae* during 2001–2010 was upraised from 26% to 39% (data from the Ministry of Public Health, Thailand). Probably related to the surge of ESBL producers, a survey of carbapenem use in Thailand during 2010–2013 showed a significant increase from approximately 2.1 to 3.1 million vials per year. However, very little is known about the molecular epidemiology and genetic characteristics of CRE in Thailand. Here we investigated the prevalence and resistance characteristics of CRE at the largest university hospital in Thailand with an emphasis on the characterization and molecular typing of CRKP.

## Materials and Methods

### Bacterial strains and antimicrobial susceptibility testing

A total of 12,741 non-duplicated clinical isolates of *Enterobacteriaceae* obtained from both in-patients (68.3%) and out-patients (31.7%) at the Faculty of Medicine Siriraj Hospital, a 2300-bed university hospital in Bangkok (Thailand), over a 2-year period (2009–2011) were investigated in this study including *K*. *pneumoniae* (38.7%), *Escherichia coli* (37.2%), *Enterobacter cloacae* (6.0%), *Proteus mirabilis* (4.2%), *Salmonella* spp. (4.1%) and other species (9.8%). All *Enterobacteriaceae* isolates were determined for their susceptibility to various antimicrobial agents by standard disk diffusion method using Kirby-Bauer disk (Oxoid, UK) according to clinical breakpoints recommended by the Clinical Laboratory Standards Institute (CLSI) [26]. Isolates that were resistant to at least a carbapenem agent were designated as CRE according to the definition recommended by the Centers for Disease Control and Prevention (available at www.cdc.gov, last updated June 29, 2015) and included for further study. In addition, isolates that were resistant to ertapenem, but not other carbapenems, and resistant to extended-spectrum cephalosporins were also included. The study isolates were confirmed by measuring minimal inhibitory concentrations (MICs) of all four carbapenems (ertapenem, imipenem, meropenem or doripenem) as well as ceftazidime and ciprofloxacin by using Etest method (bioMérieux, France) and interpreted based on the CLSI’s guideline [26]. Tigecycline was additionally tested against CRKP isolates by using Etest method and was interpreted based on the European Committee on Antimicrobial Susceptibility Testing (EUCAST)’s recommendation of S ≤1/R >2 μg/mL [27]. MIC_50_ and MIC_90_ of each tested agent were determined. Study isolates were also phenotypically characterized for the production of ESBL (using combination disk test) and carbapenemase (using modified Hodge test, MHT) by following the CLSI’s guideline [26].

### Genetic studies of beta-lactamase (*bla*) genes

Genomic DNA samples from all CRE isolates were sought by PCR sequencing for various *bla* genes including carbapenemase-related genes (*bla*_KPC_, *bla*_IMP_, *bla*_VIM_, *bla*_NDM_ and *bla*_OXA-48_) and other non-carbapenemase-related genes (*bla*_TEM_, *bla*_SHV_, *bla*_CTX-M_, *bla*_OKP_, *bla*_VEB_, *bla*_OXA_ and *bla*_AmpC_) using primers and conditions described previously [28–31]. In addition, CRKP isolates were also determined for *bla*_GES_, another member of class A carbapenemase gene, according to a published study [32].

### Expression study of outer membrane proteins

Expression of genes encoding outer membrane proteins (OmpK35 and OmpK36 porins) was genetically determined from their mRNA levels for selected CRKP isolates using real-time reverse transcription PCR. A total RNA was extracted and reversely transcribed into cDNA using High Pure Isolation Kit (Roche Diagnostics, USA) and iScript^TM^ Reverse Transcription Supermix (Bio-Rad Laboratories, USA) according to the manufacturers’ recommendations. The expression of porin-encoding genes, *ompK35* and *ompK36*, relative to *rpoB* gene was determined in triplicates by real-time PCR using specific primers and conditions as described previously [33]. Relative expression was analysed by Bio-Rad CFX manager software using 2-Δ(ΔCT) formula, where ΔCT represented the difference of cycle threshold (CT) of gene target and normalizer, and Δ(ΔCT) represented the difference of ΔCT of tested isolate and ΔCT of *K*. *pneumoniae* ATCC 13833 which was used as the control strain to determine relative expression level of both genes. The results from isolates resistant to all four carbapenems were compared to isolates resistant to only some (≤3) carbapenems. The differences of *ompK35* and *ompK36* gene expression among these two groups were determined using the non-parametric Mann-Whitney test and were considered statistically significant if *P* ≤ 0.05.

### Molecular epidemiology study

Molecular typing of CRKP isolates was identified by using both pulsed-field gel electrophoresis (PFGE) and MLST. For PFGE, chromosomal DNA of study isolates were obtained for molecular typing using a CHEF Mapper XA apparatus (Bio-Rad Laboratories) based on a *Xba*I (New England BioLabs, USA) digestion protocol [34]. A dendogram was generated by both Dice coefficient and Pearson correlations based on the unweighted pair group method using arithmetic averages (UPGMA) to determine the DNA similarities according to the Fingerprinting II Software, version 3.0 (Bio-Rad Laboratories). Clonal relatedness among isolates was defined if the Dice coefficient correlation was over 80% [35]. The clonal relationships of isolates based on MLST were sought by the similarity of an allelic profile of seven housekeeping genes to assign ST using universal primers and PCR conditions stated by the Institut Pasteur MLST (http://www.pasteur.fr/mlst).

### Ethics statement

Only bacterial isolates recovered and leftover from routine diagnostic laboratory were used in this study without a direct use of clinical specimens. Patient consents were not required. The study was ethically approved by the Siriraj Institutional Review Board, Faculty of Medicine Siriraj Hospital, Mahidol University (approval number Si 454/2009).

## Results

### CRE isolates and their resistance characteristics

Of 12,741 study isolates, 181 isolates (1.4%), including *E*. *cloacae* (*n* = 123, 67.9%), *K*. *pneumoniae* (*n* = 36, 19.9%), *E*. *coli* (*n* = 17, 9.4%) and *Citrobacter freundii* (*n* = 5, 2.8%), were shown to be resistant to at least a carbapenem and identified to be CRE. One-hurdred and forty-three isolates (79%) were recovered from urine, whilst other sources included blood, sputum and body fluids. Among CRE, twenty isolates (11.1%) were resistant to all four tested carbapenems including *E*. *coli* (*n* = 1, 5.9%), *E*. *cloacae* (*n* = 12, 9.8%) and *K*. *pneumoniae* (*n* = 7, 19.4%). Among *E*. *coli*, *E*. *cloacae*, *C*. *freundii* and *K*. *pneumoniae* CRE isolates, all isolates were resistant to ertapenem, and 4 (23.5%), 35 (28.5%), 2 (40%) and 19 (52.8%) isolates, respectively, were resistant to multiple carbapenems. MIC range, MIC_50_ and MIC_90_ as well as resistance rates of ertapenem, imipenem, meropenem, doripenem, ceftazidime and ciprofloxacin among CRE isolates are summarized in Table 1. Resistance rates to carbapenems agents other than ertapenem among CRE were 17.1–28.2%. Most isolates were also highly resistant to ceftazidime and ciprofloxacin. These CRE isolates were additionally tested for other antimicrobial agents by disk diffusion method. The results showed that they were multidrug-resistant due to a high resistance rate to several antimicrobial classes, including amikacin (46.8%), gentamicin (79.1%), netilmicin (67.4%), tetracycline (89.3%) and trimethoprim/sulfamethoxazole (87.8%). Based on phenotypic detection, ESBL production was confirmed in 138 isolates (76.2%) and only 26 isolates (14.4%) were positive for MHT.

**Table 1:** MIC range, MIC_50_, MIC_90_ (μg/mL) and resistance rate of CRE isolates (n = 181).

Antimicrobial agent	MIC range	MIC_50_	MIC_90_	% Resistance
Ertapenem	2->32	8	>32	100
Imipenem	0.25 ->32	0.5	4	17.1
Meropenem	0.125->32	1	4	28.2
Doripenem	0.125->32	1	4	19.9
Ceftazidime	0.5->256	>256	>256	98.9
Ciprofloxacin	0.016->32	>32	>32	95.6

### Genetic characterization of *bla* genes among CRE isolates

The results from the study of *bla* genes among CRE isolates are summarized in Table 2. Only seven isolates were shown to carry carbapenemase-related *bla* genes investigated in this study; *bla*_KPC_ was found in single isolates of *E*. *cloacae*, *E*. *coli and C*. *freundii*, and *bla*_IMP_ was found in four isolates of *K*. *pneumoniae*. PCR sequencing of *bla*_KPC_ demonstrated that all three isolates carried *bla*_KPC-13_ while all *bla*_IMP_-carrying *K*. *pneumoniae* had *bla*_IMP-14a_ variant. Other carbapenemase-related *bla* genes (*bla*_VIM_, *bla*_NDM_ and *bla*_OXA-48_) were not detected. A carriage rate of non-carbapenemase *bla* genes was high among CRE isolates, in which 169 (93.4%) and 158 (87.3%) isolates carried *bla*_CTX-M_ and *bla*_TEM_, respectively. Almost all isolates of *E*. *cloacae* and *C*. *freundii* carried both *bla*_CTX-M_ and *bla*_TEM_ genes. *bla*_SHV_ was most prevalent in *K*. *pneumoniae* (n = 33, 91.7%), while *bla*_VEB_ was found only in *E*. *cloacae* (n = 16, 13.7%) and *C*. *freundii* (n = 1, 20%), and *bla*_OKP_ was found only in *K*. *pneumoniae* (n = 2, 5.6%). *bla*_AmpC_ was most commonly detected in *E*. *cloacae* (n = 118, 95.9%), and among them, 114 isolates (96.6%) were demonstrated to be the EBC (MIR/ACT) family of *bla*_AmpC_ gene. Carbapenemase-unrelated *bla*_OXA_ was found in 34 isolates (18.8%), except for *E*. *coli*.

**Table 2:** Genetic study of *bla* genes among CRE isolates.

Organism (n)	No. (%) of isolate												
	Carbapenemase-related *bla* gene						Carbapenemase-unrelated *bla* gene							
	KPC	GES	IMP	VIM	NDM	OXA-48	TEM	SHV	CTX-M	OKP	VEB	AmpC	OXA^a^
*E*. *cloacae* (123)	1 (0.8)	ND	0	0	0	0	119 (96.7)	11 (8.9)	122 (99.2)	0	16 (13.7)	118 (95.9)	28 (22.8)
*K*. *pneumoniae* (36)	0	0	4 (12.1)	0	0	0	23 (63.9)	33 (91.7)	30 (83.3)	2 (5.6)	0	4 (11.1)	5 (13.9)
*E*. *coli* (17)	1 (5.9)	ND	0	0	0	0	11 (64.7)	4 (23.5)	13 (76.5)	0	0	2 (11.8)	0
*C*. *freundii* (5)	1 (20)	ND	0	0	0	0	5 (100)	2 (40)	4 (80)	0	1 (20)	1 (20)	1 (20)
**Total (181)**	**3 (1.7)**	**0**	**4 (2.2)**	**0**	**0**	**0**	**158 (87.3)**	**50 (27.6)**	**169 (93.4)**	**2 (0.01)**	**17 (9.4)**	**125 (69.1)**	**34 (18.8)**

^*a*^ *bla*_OXA_ genes were determined by DNA sequencing to encode non-carbapenemase-related OXAs.

ND, not determined.

### Characterization of CRKP isolates

Compared to CRE isolates of other species, CRKP isolates had the highest percentage of isolates that were resistant to multiple carbapenems (52.8%), suggesting its significance as CRE. We thus further characterized all CRKP isolates for their resistance genotype and molecular epidemiology. From this survey, a total of non-duplicated 4,929 isolates of *K*. *pneumoniae* were recovered from various clinical specimens. ESBL phenotype was confirmed for 2,311 isolates (46.9%). Thirty-six isolates (0.7%) were identified to be CRKP. These CRKP isolates were recovered from patients with an age range from 2-month to 95-year old (median = 67.5 years old), and 22 patients (61.1%) were male. Most isolates (77.7%) were from patients hospitalized in various wards (medicine 30.6%, surgery 19.4%, pediatrics 8.3% and intensive care units 19.4%) and the rest were out-patients.

Evaluation for ESBL production, MHT and MIC values of all four carbapenems and other tested drugs for each CRKP isolate are shown in Table 3. They had high MIC_50_/MIC_90_ values against all carbapenems (16/>32, 1/32, 2/32 and 2/32 μg/mL for ertapenem, imipenem, meropenem and doripenem, respectively). All isolates were resistant to ertapenem, while 7 (19.4%), 16 (44.4%) and 17 (47.2%) isolates were resistant to imipenem, doripenem and meropenem, respectively. Seven isolates that were resistant to imipenem were also resistant to all other tested carbapenems. Thirty-one (86.1%) and 35 (97.2%) isolates were resistant with high MIC values to ciprofloxacin and ceftazidime, respectively. For tigecycline, 11 isolates (30.6%) were intermediate and 8 isolates (22.2%) were resistant. Among CRKP, 28 isolates (77.8%) were ESBL-positive, and 9 isolates (25%) were MHT-positive. Most CRKP harbored multiple *bla* genes, especially in the TEM, SHV and CTX-M families (Tables 2 and 3). *bla*_SHV_ was found in most CRKP isolates (91.7%). Twenty-eight isolates harbored at least one ESBL genes (SHV-2, SHV-12 and/or CTX-M-15). The most common ESBL-related *bla* gene was CTX-M-15, carried by 26 (72.2%) CRKP isolates. Four CRKP isolates (No. 3, 22, 38, 40) had *bla*_IMP-14a_ but the rest did not carry any carbanemase-related *bla* genes investigated in this study, including *bla*_GES_. All *bla*_IMP-14a_-carrying isolates co-harbored *bla*_OXA-10_, and each isolate may also harbor additional one to three *bla* genes.

**Table 3:** ESBL production, MHT results, MIC values and *bla* genotypes of CRKP isolates/

Isolate No.	ESBL	MHT	MIC (μg/mL)^a^							*bla* genotype ^b^
			ETP	IMP	MEM	DOR	CAZ	CIP	TGC	
1	+	-	16	1	4	2	128	>32	2	TEM-1, SHV-11, CTX-M-15
2	+	-	8	2	0.5	0.5	>256	1	1	SHV-11
3	-	+	>32	>32	32	>32	>256	1	1	IMP-14a, OKP-B-13, OXA-2, OXA-10
4	+	-	16	2	2	2	>256	>32	4	TEM-1, SHV-12, CTX-M-15
5	-	-	>32	>32	32	32	>256	0.064	0.5	TEM-1, SHV-11, CTX-M-15
6	+	+	8	1	2	2	>256	>32	4	TEM-1, SHV-12, CTX-M-15
7	+	-	>32	1	4	2	>256	>32	2	TEM-1, SHV-11, CTX-M-15
8	-	-	>32	1	8	4	>256	>32	2	TEM-1, SHV-11, CTX-M-15
9	+	+	16	1	2	2	>256	>32	2	SHV-2, CTX-M-15
10	+	-	2	0.25	0.25	0.25	>256	>32	0.5	TEM-1, SHV-11, CTX-M-15
11	+	-	>32	1	4	2	>256	>32	0.5	TEM-1, SHV-11, CTX-M-15
12	+	-	>32	2	8	8	>256	>32	2	TEM-1, SHV-11, CTX-M-15
13	+	-	8	0.5	0.5	0.5	>256	>32	1	SHV-12, CMY-2
14	+	-	16	0.5	1	1	>256	>32	2	TEM-1, SHV-11, CTX-M-15
15	+	-	>32	8	>32	32	>256	>32	4	SHV-1, CTX-M-15
16	+	-	8	0.5	1	0.5	32	>32	2	TEM-1, SHV-1, CTX-M-15
17	+	-	16	1	2	2	>256	>32	2	TEM-1, SHV-28, CTX-M-15
18	+	+	>32	4	16	8	>256	>32	1	SHV-11, CTX-M-15
19	+	-	>32	1	4	4	>256	>32	1	TEM-1, SHV-11, CTX-M-15
20	+	-	16	1	2	2	>256	32	2	SHV-1, CTX-M-15
21	+	-	>32	2	8	4	>256	>32	1	TEM-1, SHV-11, CTX-M-15
22	-	+	8	0.5	4	8	>256	1	1	IMP-14a, OXA-10
23	+	-	8	0.25	0.5	0.5	>256	>32	4	TEM-1, SHV-11, CTX-M-15
24	+	-	>32	32	4	4	>256	>32	1	TEM-1, SHV-11, DHA-1
26	+	-	2	0.25	0.25	0.25	>256	>32	4	TEM-1, SHV-11, CTX-M-15, DHA-1
27	+	-	8	0.5	1	1	64	>32	4	TEM-1, SHV-1, CTX-M-15
28	+	-	4	0.25	0.5	0.5	>256	>32	1	TEM-1, SHV-12
30	-	+	2	0.125	0.25	0.125	>256	4	2	TEM-1, SHV-12, CTX-M-15
31	-	-	>32	32	32	16	>256	>32	1	SHV-11, DHA-1
32	+	-	8	1	1	0.5	>256	>32	2	TEM-1, SHV-11, CTX-M-15
33	+	+	>32	2	4	8	4	0.064	1	OKP-B-13, OXA-2
34	+	-	>32	8	32	16	>256	>32	1	TEM-1, SHV-11, CTX-M-15
35	+	-	2	0.25	0.25	0.25	>256	>32	4	TEM-1, SHV-11, CTX-M-15
36	-	+	2	2	2	4	128	32	1	IMP-14a, SHV-11, OXA-10
38	+	-	>32	1	8	4	>256	>32	8	SHV-11
40	-	+	2	2	2	4	>256	8	1	IMP-14a, SHV-11, CTX-M-15, OXA-10

^*a*^Drug abbreviation: ertapenem (ETP), imipenem (IMP), meropenem (MEM), doripenem (DOR), ceftazidime (CAZ), ciprofloxacin (CIP), tigecycline (TGC)

^*b*^ *bla*_SHV-2_, *bla*_SHV-12_ and *bla*_CTX-M-15_ are ESBL-related genes, and *bla*_IMP-14a_ is a carbapenemase-related gene.

### Expression of *ompK35* and *ompK36* genes

Seventeen CRKP isolates were selected for further evaluation of *ompK35* or *ompK36* gene expression including seven isolates that were resistant to all four carbapenems (No. 3, 5, 15, 18, 24, 31, 34) and ten isolates that were partly resistant to some carbapenems. Among isolates that were resistant to all carbapenems, most of them had high MIC values against carbapenems, but only one isolate (No. 3) carried a carbapenemase gene (*bla*_IMP-14a_). Amount of mRNAs expressed from *ompK35* and *ompK36* genes encoding major OMP porins associated with entry of carbapenems were quantitated by real-time reverse transcription PCR and calculated as fold expression in relative to the control strain. Most isolates, comparing between isolates resistant to all four carbapenems and isolates resistant to some (≤3) carbapenems, showed a various degree of decreased expression of either *ompK35* or *ompK36* gene, or both, except for isolates No. 20 and 33 in which expression of both genes were slightly increased (Table 4). Comparing between isolates that were resistant to all four carbapenems and isolates that were resistant to some carbapenems, there was no significant difference in *ompK35* expression (*P* = 0.96). However, isolates resistant to all four carbapenems showed statistical significance in *ompK36* expression when compared to isolates resistant to some carbapenems (*P* = 0.01). According to carbapenem MIC values shown in Table 3, changes in *ompK35* expression was not significant for resistant isolates comparing to non-resistant isolates for imipenem (*P* = 0.96), meropenem (*P* = 0.52) and doripenem (*P* = 0.52). However, a decrease of *ompK36* expression was statistically associated with isolates resistant to imipenem (*P* = 0.01), meropenem (*P* = 0.05) and doripenem (*P* = 0.05).

**Table 4:** Expression of *ompK35* and *ompK36* genes among selected CRKP isolates.

Isolate No.	Susceptibility^a^				Relative fold expression^b^	
	ERT	IMP	MEM	DOR	*ompK35*	*ompK36*
Isolates resistant to all 4 carbapenems						
3	R	R	R	R	-2.36 ± 0.03	-7286.85 ± 0.01
5	R	R	R	R	-1.73 ± 0.11	-1429.15 ± 0.01
15	R	R	R	R	-5.43 ± 0.01	-4336.76 ± 0.01
18	R	R	R	R	-5.43 ± 0.02	-18.96 ± 0.01
24	R	R	R	R	-2.15 ± 0.04	-5.31 ± 0.02
31	R	R	R	R	-41.00 ± 0.01	-1.42 ± 0.06
34	R	R	R	R	-122.60 ± 0.01	-4166.33 ± 0.01
Isolates resistant to ≤3 carbapenems						
2	R	I	S	S	4.17 ± 0.65	-5.03 ± 0.02
9	R	S	I	I	1.12 ± 0.09	-2435.90 ± 0.01
10	R	S	S	S	-129.68 ± 0.01	1.21 ± 0.13
16	R	S	S	S	-37.97 ± 0.01	-4.41 ± 0.01
20	R	S	I	I	1.45 ± 0.19	2.09 ± 0.20
23	R	S	S	S	-297.80 ± 0.01	1.44 ± 0.05
28	R	S	S	S	-2.85 ± 0.03	-9.61 ± 0.01
30	R	S	S	S	-257.40 ± 0.01	-1.09 ± 0.04
33	R	I	R	R	1.43 ± 0.13	1.43 ± 0.18
35	R	S	S	S	-177.70 ± 0.01	-1.15 ± 0.06

^*a*^Susceptibility to carbapenems was interpreted as S (susceptible), I (intermediate) and R (resistant) according to MIC values shown in Table 3 based on CLSI guideline [26].

^*b*^Expression of *ompK35* and *ompK36* was normalized to *rpoB* expression and shown in relative to the expression of *K*. *pneumoniae* ATCC 13883.

### Molecular epidemiology of CRKP isolates

**Fig 1:**
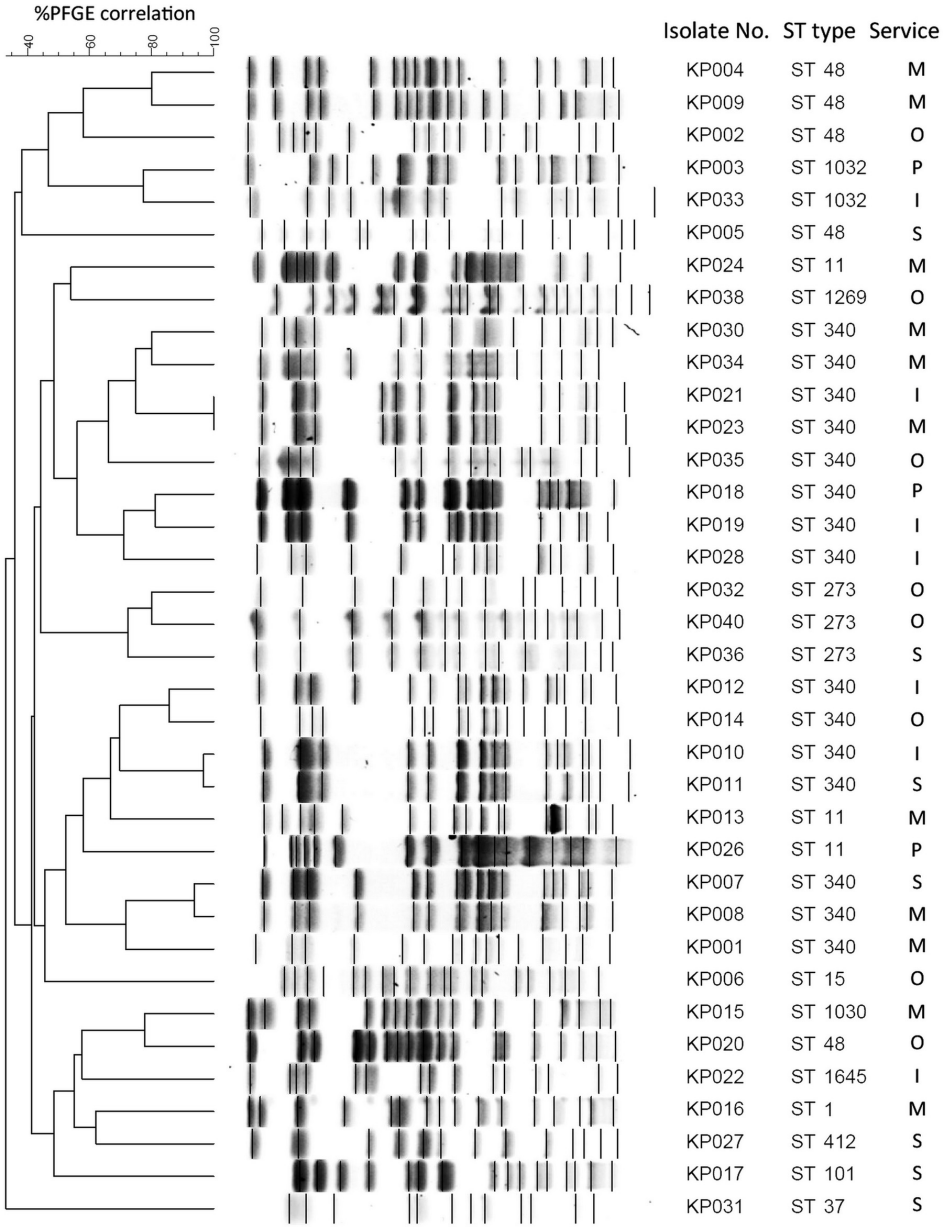
Molecular epidemiology of CRKP isolates. All CRKP isolates were determined for their clonal relatedness based on PFGE and MLST typing. Isolates from different hospital services (O, out-patient; M, medicine; S, surgery; P, paediatrics; I, intensive care units) were compared for PFGE patterns and ST types.

Clonal relatedness of all CRKP isolates was determined by two typing methods, PFGE and MLST, as shown in Fig 1. According to the PFGE typing, these CRKP isolates were belonged to several clones and no more than two isolates in each PFGE type appeared in the same clone. MLST typing, however, demonstrated that the sequence type ST340 was predominant (41.7%), followed by ST48 (13.9%), ST11 (8.3%), ST273 (8.3%), ST1032 (5.6%) and a single isolate of STs 1, 15, 37, 101, 412, 1030, 1269, 1645. CRKP ST340 isolates were identified from both in- and out-patients, and appeared to spread in intensive care units (71.4% of isolates) and medicine wards (45.5% of isolates). The ST1645 isolate was a novel type designated by this study and its DNA sequences were deposited to the Institut Pasteur MLST database for *K*. *pneumoniae* (available at http://www.pasteur.fr/mlst). Many isolates with the same STs were clustered together by PFGE typing, including ST340 isolates (Fig 1). Most of ST48 isolates (except isolate No. 20) were in the same PFGE cluster, and closely related to ST1032. ST340 isolates were also partly related to ST11 and ST273 isolates.

## Discussion

A worrisome problem of multidrug-resistant bacteria is expanding worldwide, including in the Asia-Pacific region. Previous surveys showed that the rates of ESBL-producing *Enterobacteriaceae* in Thailand were approximately 40–50% and were relatively high comparing to other countries in the Asia-Pacific region [36], [37]. However, the prevalence of CRE in Thailand is not clearly known. A previously published study demonstrated that carbapenem non-susceptible *Enterobacteriaceae* in Thailand was approximately 0.4% among isolates from intraabdominal infection, bloodstream infection and nosocomial pneumonia [37]. The overall prevalence of CRE in Thailand remained low (1.4%) during our study period, while the rate of ESBL-related *bla* gene carriage among CRE was very high. MHT was determined for the detection of carbapenemase production [26]. Our results, however, showed that MHT was not well correlated with the detection of CRE as the test was positive for 26 isolates (14.4%) and, among these isolates, only three and four isolates were positive for class A (*bla*_KPC_) and class B (*bla*_IMP_) carbapenemases, respectively. Many studies have reported that phenotypic-based MHT often yielded low sensitivity and specificity for detection of carbapenemases, especially for class B carbapenemases [38], [39]. Besides the possibility of false-positive result due to a low reliability of MHT, other mechanisms may play a role in carbapenem resistance among these isolates. For example, a majority of CRE isolates in this study were *E*. *cloacae* but only one isolate of carbapenem-resistant *E*. *cloacae* was identified to carry a carbapenemase gene. At this center, approximately 16.1% of *E*. *cloacae* were identified as CRE, whereas only 0.7% of *K*. *pneumoniae* were CRE. *E*. *cloacae* has been shown to possess multiple mechanisms other than carbapenemase production that may confer carbapenem resistance, especially AmpC hyperproduction and decreased porin expression [40]. Therefore, MHT has a very limited role in the detection of CRE and its result should be carefully interpreted.

Although KPC-producing *Enterobacteriaceae* have been reported to spread rapidly in the last decade, the prevalence in Thailand remains very low. This study demonstrated that the rate of *bla*_KPC_-carrying isolates was only 0.02% among *Enterobacteriaceae* and 1.7% among CRE isolates. We also reported previously from this survey that it was the first emergence of *bla*_KPC_ in Thailand [41]. More interestingly, none of them were found in *K*. *pneumoniae* isolates, the most common host of *bla*_KPC_, and they were all characterized as a novel variant designated *bla*_KPC-13_. The *bla*_IMP-14a_ has recently been reported among the emerging *Enterobacteriaceae* isolates carrying New Delhi metallo-beta-lactamase (NDM)-1 in Thailand [42], but has not been reported from other countries. In addition, this gene has only been detected in *K*. *pneumoniae*. This may indicate a local spread of *bla*_IMP-14a_ among *K*. *pneumoniae* in Thailand. It is noteworthy that only 3.9% of CRE isolates were detected for important carbapenemase gene families (*bla*_KPC_ and *bla*_IMP_). Therefore, the dissemination of carbapenemase genes among CRE in Thailand is very low and unique from other countries, and other mechanisms would play a more important role in carbapenem resistance among *Enterobacteriaceae*.

Most CRKP isolates were highly resistant to ceftazidime and ciprofloxacin. It should even more concerned for susceptibility to tigecycline. Many previous reports showed that tigecycline was active against CRE, but in our study only 47.2% of CRKP isolates remained susceptible to tigecycline. This suggests that CRKP may cause a serious difficulty to find appropriate treatment and a close monitoring for the spread of CRKP is highly recommended. The extensive genetic characterization of *bla* genes among CRKP demonstrated that most isolates harbored multiple *bla* genes, especially ESBL-related genes. Among isolates carrying ESBL genes, the *bla*_CTX-M-15_ was most commonly found, followed by *bla*_SHV-12_. However, the results of ESBL phenotypic test were not completely correlated with the genotypic study. Similarly, the detection of carbapenemase-related *bla* gene was not well related to the MHT results and carbapenem MIC values of these CRKP isolates. Therefore, the MHT result has a limitation to predict for the production of carbapenemases, as well as the susceptibility levels of CRKP against carbapenem agents, and the detection of *bla* carbapenemase genes may only partially explain carbapenem resistance among CRE. Due to a very limited choice of treatment, results of antimicrobial susceptibility testing should be carefully considered as a therapeutic guidance against CRKP.

Multiple mechanisms can contribute to carbapenem resistance among CRKP. Besides the production of carbapenemases, the loss or reduction of porins, OmpK35 and/or OmpK36, has been shown to correlate with increased carbapenem MICs [14–17]. Seventeen CRKP isolates, including seven isolates resistant to all four carbapnems and ten isolates resistant to some (≤3) carbapenems, were selected to study the expression of porin proteins. Only one of isolates resistant to all four carbapenems carried a carbapenemase gene, *bla*_IMP-14a_. Most isolates, except two, demonstrated a decrease in expression of *ompK35* or *ompK36* gene, or both, at varying degrees. A reduction of *ompK35* expression did not show a significant difference among isolates resistant to all four carbapenems and isolates resistant to some carbapenems, and did not correlate with levels of increased MIC values of each carbapenem among these isolates. However, it was revealed that isolates resistant to all four carbapenems had a significant decrease of *ompK36* expression comparing to isolates resistant to some carbapenems. In addition, a decrease of *ompK36*, but not *ompK35*, expression was statistically associated with individual carbapenem resistance (imipenem, meropenem and doripenem) among CRKP isolates. This suggests that a decrease of *ompK36* expression may be involved in carbapenem resistance with a higher magnitude than does a decrease of *ompK35* expression. In addition, it supports previous studies that porin deficiency contributes with a lesser role in carbapenem resistance comparing with the production of beta-lactamases [17], [43].

The PFGE study indicated that various PFGE patterns of CRKP were present and there was no predominated clone. Thus, multiple CRKP clones were disseminated through different hospital services. However, the MLST study revealed that ST340 was the most significant clone among CRKP isolates in this study, with an evidence of its community spread since it was also recovered from ambulatory patients. A half of CRKP isolates were members of the drug-resistant *K*. *pneumoniae* CC258 (ST340, 41.7% and ST11, 8.3%). Isolates of CC258 have been shown to be associated with the carriage of KPC gene and international spread in many countries, including North America, Latin America and Asia, and mostly ST11 and ST258 are the common types [20], [22], [24]. ST258 is currently known to be an epidemic clone of KPC-producing *K*. *pneumoniae*. Interestingly, KPC gene was not detected in our CRKP isolates and none of isolates were ST258. The epidemiologic information of CRKP in Thailand is unknown. We here report that ST340, a member of CC258, is the most common type among CRKP isolates in Thailand. ST340 clone was sporadically reported, e.g. from Israel and Russia [25], [44], but has never represented the predominant clone. In addition, ST48, the second most common clone in this study, has also rarely been reported among CRKP isolates. Several other ST types were found among CRKP isolates, including the novel ST1645 clone. An isolate of ST101 was found in this study. This clone has recently been reported to be the predominant clone of *K*. *pneumoniae* carrying beta-lactamase genes among companion animals in Italy, in which CC258 isolates were also identified from this source [45]. In addition, ST101 clone was associated with NDM-1-producing *K*. *pneumoniae* in many regions [46]. This may also suggest a possibility of companion animals as vector to transfer resistant genes to human and further study should be considered. Our study also demonstrated that PFGE and MLST studies were in partial concordance. Isolates in the CC258, ST340 and ST11, were clustered more closely based on PFGE patterns, while non-CC258 members were more distant. These data hence suggest that CRKP isolates in Thailand have a unique epidemiologic characteristic and appear to be widespread in both in- and out-patient services.

In conclusion, we report an extensive survey for the emergence of CRE at the largest university hospital in Thailand. The prevalence of CRE was low but should be closely monitored. Most CRE isolates harboured multiple *bla* genes. Only a few isolates harboured a carbapenemase gene, *bla*_KPC-13_ or *bla*_IMP-14a_, which were particularly found in Thailand. A decreased porin expression in selected CRKP isolates was not directly concordant with levels of increased carbapenem MICs. Therefore, further investigation is required to better understand the mechanisms that contribute to carbapenem resistance among CRE isolates. The PFGE and MLST studies revealed that multiple clones of CRKP were widespread. CC258, especially ST340, was the most predominant clone. Our findings represent the novel epidemiologic data of CRKP in Thailand and ones should be an alert for continuous surveillance of CRE and CRKP in this region to promptly control these highly resistant bacteria.
